# Role of rat sarcoma virus mutations in cancer and potential target for cancer therapy

**DOI:** 10.2144/fsoa-2019-0045

**Published:** 2020-02-21

**Authors:** Sanjana S Murdande

**Affiliations:** 1 ROSS University School of Medicine, Lloyd Erskine Sandiford Center, St Michael BB11039, Barbados

**Keywords:** anticancer, cancer, gene, gene therapy, mutation, oncogene, personalized medicine, RAS, RAS-mitogen, target

## Abstract

The prevalence of oncogenic rat sarcoma virus (RAS) mutations has made RAS a popular target for cancer therapies. Significant discoveries have been reported regarding cancer molecular biology following the study of RAS mutations. These discoveries are integral in shaping the era of targeted cancer therapy, with direct targeting of RAS or downstream RAS effectors, such as Grb2 and MAPK a possibility. Novel agents such as farnesyltransferase directly bind and sequester RAS. While these new agents and approaches have shown promise in preclinical and clinical studies, the complexity of RAS signaling and the potential for robust adaptive feedback continue to present substantial challenges. Therefore, the development of targeted therapies will require a detailed understanding of the properties and dependencies of specific cancers to a RAS mutation. This review provides an overview of RAS mutations and their relationship with cancer and discusses their potential as therapeutic targets.

## Lay abstract

The widespread prevalence of cancer has focused research attention on the discovery and development of newer therapies. Significant discoveries regarding genetic mutations have provided new opportunities for development of targeted cancer therapies. In this review, mutations in the rat sarcoma virus protein are discussed along with their potential as targets for drug development.

A contributing factor to numerous human cancers is the rat sarcoma virus (RAS) protein. Some of the most frequently detected oncogene alterations, both in animal and human cancers, are mutations in the *RAS* oncogene family [1]. The RAS family of small GTPases consists of four highly related members: K-Ras 4A, K-Ras 4B, H-Ras and N-Ras. The proteins are made in the cell cytosol and located at the inner leaflet of the plasma membrane, where they are involved in signal transduction through multiple partners/effectors [2]. A small GTPase converts between an active, GTP-bound conformation and an inactive, GDP-bound conformation. RAS regulates multiple cellular processes such as proliferation, motility and gene expression [2]. It is foremost an oncogene that, when mutated, results in uninhibited cell growth. A point-mutated RAS keeps the molecule in an active, GTP-bound state, causing abnormal cell signaling. The RAS pathway begins with a RTK enzyme, which upon stimulation from a molecule such as a growth factor or hormone, cross-phosphorylates itself. A series of molecules then become phosphorylated in a cascade of events. Active RAS controls PI3K and AKT as well as MAPK. The MAPK cascade consists of serine/threonine kinases, which convert extracellular molecules such as growth factors, hormones, tumor-promoting substances and differentiating factors, into intracellular signals for regulating cell proliferation, differentiation and survival [2]. The phosphorylated Raf1, an MAP3K, in turn activates MEK [3]. The guanine nucleotide exchange factors (GEFs) carry GTP to the RAS molecule, which becomes activated upon the changing of GDP for GTP [4]. The *RAS-GEF* gene was first discovered in 1992 [5]. RAS mutations also cause an accumulation of reactive oxygen species, leading to DNA damage in cells. The cell checkpoint feature that typically inhibit cells with DNA damage from proceeding to mitosis does not function in the light of the RAS mutation, allowing genetically injured cells to proceed through the G1 and G2 phases of cell growth. The cells that lack the *p53* tumor suppressor gene are particularly affected by these consequences of RAS mutations, as *p53* functions against the *RAS* oncogene [6]. It has been determined that RAS mutations are more prevalent in certain cancers but less likely associated with others, such as breast cancer [3]. Instead, most breast cancers, including ductal and lobular, are characterized by overexpression of the RAS protein [3].

The *H-RAS* and *K-RAS* genes were the first to be identified in rats from the Harvey sarcoma virus and the Kirsten sarcoma virus, respectively [7,8]. Therefore, the term RAS is derived from rat sarcoma. The *N-RAS* gene was discovered in human neuroblastoma cells in the 1980s. The *K-RAS* gene stimulates a cascade of proteins that activates cellular anti-apoptotic activity, therefore promoting cell growth. The PKB/AKT pathway is one such protein cascade that, when activated, phosphorylates the Bad protein, which in turn inhibits cellular apoptosis [3]. However, the mutant *K-RAS* gene produces high levels of the *P19^ARF^* tumor suppressor gene that activates the *p53* tumor suppressor gene, which leads to cellular apoptosis. Thus, *K-RAS* has opposing functions. *K-RAS* is activated in a large number of cancers, while *H-RAS* and *N-RAS* are only activated in certain cancers such as melanomas and carcinomas [9]. Colon, pancreatic and lung cancers are prime examples for *K-RAS* mutations. It has been determined through research using carcinogen-induced tumor mouse models that both *K-RAS* and *H-RAS* have nearly equivalent tumor-causing properties [4]. In a colon cancer mouse model, *K-RAS*, and not *N-RAS*, was able to proliferate epithelial colon cancer [10].

## RAS protein

All mammalian cells express three closely related RAS proteins: *H-RAS*, *K-RAS* and *N-RAS*. These promote oncogenesis when they are mutationally activated at codon 12, 13 or 61 [8]. Although the isoforms exhibit a high degree of similarity, *K-RAS* has been shown to be the most frequently mutated isoform in most cancers; for example, 90% of pancreatic tumors harbor *K-RAS* mutations. By contrast, *N-RAS* mutations are strongly associated with hematopoietic tumors [9]. These proteins are GTPases that function as molecular switches in regulating pathways that are responsible for proliferation and cell survival [10].

In a study performed using human T-cells, it was determined that Tax-positive cells had high levels of RAS-GTP [11]. RAS-GTP activates the anti-apoptotic activity of Tax by interfering with the mitochondrial apoptotic pathway. Tax, armed with this anti-apoptotic ability, survives to convert normal cells into cancer cells and promote abnormal cell growth. In this same study, the RAS antagonist farnesyl thyosalicylic acid was applied to the T-cells. Farnesyl thyosalicylic acid deactivates RAS-GTP into RAS-GDP. It was apparent that the apoptotic activity of Tax increased in correlation with more significant levels of inactive RAS-GDP [11].

Tax interacts with and inhibits GAP, which is a RAS GTP-ase activating protein. The GTP-ase hydrolyzes RAS-GTP (active form) into RAS-GDP (inactive form). The inactivity of GAP results in a buildup of RAS-GTP. The accumulation of active RAS-GTP promotes the anti-apoptotic nature of Tax, thereby making cells more resistant to chemotherapy. The Tax protein is a significant reason why cancerous T-cells are resistant to chemotherapy treatment, due to the apoptotic-resistance factor. Approximately 30% of all human tumors express a mutant form of RAS that is locked in the active form as result of being insensitive to GAPs [12]. Furthermore, GAPs act exclusively on normal, nononcogenic RAS proteins in RAS-transformed cells. This leads to the buildup of active RAS-GTPases. An additional accumulation of GEFs allows for the removal of GDP and the addition of GTP to RAS, which can then hyper-activate downstream proteins in its pathway [13]. Phosphorylated, activated ERK protects human T-cells with Tax from apoptosis. In a study carried out on human T-cells, it was reported that the Tax-expressing cells have high levels of RAS-GTP (active form) and that RAS-GTP contributes to Tax-mediated apoptosis protection. Treatment of cells with a RAS antagonist decreased their apoptotic resistance and treatment was also accompanied by a reduction of both RAS-GTP and phosphorylated ERK1/2 [11]. The results in this study indicated RAS as a possible target for adult T-cell leukemia and lymphoma therapy [11].

## *RAS* oncogene & human cancer

Numerous other hematopoietic disorders feature deregulated RAS signaling, which is caused by irregular GAPs that abnormally change RAS-GTP into RAS-GDP. The various cancers related to RAS mutations include non-small-cell lung carcinoma, colorectal carcinoma and pancreatic carcinoma. For *K-RAS*, they are caused by a substitution of the amino acid glycine by aspartate or valine at codon 12 and by aspartate at codon 13 on the exon 1 fragment of the *K-RAS* gene. The *K-RAS* mutations are present in a significant amount of colorectal cancers, while *N-RAS* mutations are only present in a small amount of cancers. Both *K-RAS* and *N-RAS* mutations affect codons 12, 13 and 61 in colorectal cancer [14]. The *RAS* mutations at these sites commonly inhibit binding between RAS and GAPs, thus preventing GTP hydrolysis [4]. In mice, it has been shown that acute myeloid leukemia may be induced through oncogenic *K-RAS* as well as a deficiency of the *Nf1* gene [15]. Furthermore, RASA3, which is a Ras-GTPase activating gene located at the plasma membrane close to RAS, has been shown to cause various cancers in mice and humans when it is inactivated, causing a buildup of active RAS-GTP [16]. These cancers include glioblastoma, squamous-cell carcinoma and abdominal cavity sarcoma. In a study done in oral squamous cell carcinoma cells, it was confirmed that fibroblast activating protein is expressed and ERK signaling is a pathway that stimulates cell cycle signaling to promote cell proliferation [17]. ERK1 and ERK2 are important proteins in the RAS pathway that, when activated, signal to melanoma growth genes and proteins to activate. The cellular apoptosis susceptibility (CAS) protein, located on the 20q13 region of the chromosome, is overexpressed in melanoma [18]. The RAS pathway is responsible for the phosphorylation and activation of CAS in melanoma and functions in the distribution of phosphorylated CAS in the cellular cytoplasm. In a study done to test the relation between the CAS and the RAS pathway, the B16F10 melanoma cell line overexpressing RAS was used [18]. The results of the study concluded that melanoma cells featuring *RAS* mutations had an increase in phosphorylated, active CAS protein distribution to the cytoplasm of the cells. The phosphorylation of the ERK protein was regulated by CAS as well [18]. While many treatment options for malignant melanoma are short lived, targeting RAS downstream pathway proteins and related melanoma growth proteins such as CAS should be further studied [18]. In a study conducted with human fibroblasts transfected with *H-RAS*V12 (mutation at position 12 replacing amino acid glycine with valine) expressing cells, premature senescence (loss of cells power of division and growth) is associated with cellular redox imbalance as well as with altered post-translation protein modification [19].

Ovarian cancers of the epithelial tissue form are linked to *RAS* mutations as well. There is evidence of *K-RAS* mutations, varying in the degree of prevalence, in ovarian carcinomas and borderline tumors, varying in the degree of prevalence in these cancers. Studies suggest that K-*RAS* mutations are a common early occurrence in ovarian tumor formation [20]. *K-RAS* mutations then stimulate the RAS downstream cellular signaling pathway. Type 1 ovarian cancers typically exhibit *K-RAS* mutations, while type 2 ovarian cancers do not exhibit *RAS* mutations but are associated with mutations in TP53 gene [21]. Hepatocellular carcinoma is another cancer of interest with regards to RAS mutation and the RAS pathway dysfunction. Numerous downstream molecules in the RAS pathway have been found to be activated in hepatocellular carcinoma [22]. A significant amount of cases of hepatocellular carcinoma are attributed to a *B-Raf* gene mutation or deletion, which subsequently activates an oncogenic RAS, as well as *K-RAS*, *H-RAS* and *N-RAS* mutations [4].

## *RAS* mutations as a target for cancer therapy

The prevalence of oncogenic *RAS* mutations has made RAS a popular target for cancer therapies. Significant discoveries are reported regarding cancer molecular biology as a result of the study of oncogenic *RAS* mutations. These have been integral in shaping the era of targeted cancer therapy. *RAS* mutations are one of the most common oncogenic drivers in human cancer and there are ongoing efforts to find a clinically effective Ras inhibitor [23]. RAS genes are the some of the most commonly mutated oncogenes in cancer, but effective therapeutic strategies to target RAS-mutant cancers have proven elusive [24]. The direct inhibition of RAS proteins has proved difficult, which has led researchers to test alternative strategies that target RAS effectors [24]. RAS is a small guanine nucleotide-binding protein that regulates the state of signal transduction pathways central to cell growth, proliferation and survival [25,26]. It switches between active and inactive signaling states, depending on if it is in GTP- or GDP-bound state, respectively. A structural study has taught that guanosine nucleotides dictate these states primarily by altering the conformation of two dynamic loops within the RAS proteins called switch I and switch II [27]. Furthermore, the most common *RAS* mutations occur at codons 12, 13 and 61. These mutations surround the guanine nucleotide-binding pocket suggesting that the most common mechanism by which activating *RAS* mutations function is to influence aspects of how nucleotides interact with RAS, be it binding, exchange, hydrolysis or control of protein dynamics [27]. If the mechanisms that underlie the functional classes of *RAS* mutations can be defined, it follows that strategies to address these classes may be customized [27].

The RAS-MAPK pathway, however, proves evasive to numerous drug treatments, as there are many molecules related to RAS and effective targeting can prove difficult. Drug discovery efforts have been primarily focused in four areas: direct inhibition of RAS, blocking enzymes that catalyze post-translational modifications of RAS and that are essential for membrane targeting, developing inhibitors of downstream effectors and searching for synthetic lethal interactors of mutant RAS [28,29]. Furthermore, patient responsiveness to numerous therapies can be transient.

Existing treatments include the drug Salirasib, which directly inhibits RAS by removing the molecule from the cellular membrane to which it is bound. In addition to this removal, the RAS downstream pathway and related pathways are also inhibited and the amount of intracellular RAS levels decline [4]. Despite the existence of treatments such as Salirasib, unique approaches to targeting RAS are still being studied. Drug combination therapy is one such approach that is being tested as an efficient treatment. One study has demonstrated that the combination of poly(adenosine diphosphate-ribose) polymerase (PARP) inhibitors as well as MEK inhibitors is an effective drug combination therapy [8]. Both inhibitors function harmoniously to induce cellular apoptosis in tumors exhibiting *RAS* mutations. The effectiveness of the PARP and MEK drug combination therapy was tested using ovarian cancer cell lines. It was further determined through the study that the presence or absence of *p53* mutations in the cancerous cells did not alter the efficacy of PARP and MEK drugs in combination [8]. Trametinib, which is a MEK inhibitor, proved to be more successful in treating cancer compared with traditional chemotherapy [4]. Other MEK inhibitor drugs include Cobimetinib, Cetuximab, Selumetinib and Pimasertib. These drugs are being tested in combination with each other for efficacy. Selumetinib and Cetuximab were tested together in patients with *K-RAS*-related colorectal cancer with good results [4]. In other treatment methods, competitive inhibitors are used to counteract the downstream proteins of the RAS pathway, such as MAPK, Raf kinase and phosphoinositide 3-kinase. Proteins such as the receptor tyrosine kinase EGF activate oncogenes in the Ras signaling pathway, which subsequently express proteins such as Grb2. EGF persists in many breast and ovarian cancers [30]. Grb2 is a downstream effector in the Ras signaling pathway that is a potential antitumor drug therapy target. Grb2 may be inhibited from either its SH2 or SH3 domains [30]. Additionally, isoprenyltransferases are targeted through anticancer treatments, as these molecules are essential in the binding of RAS to cellular membranes. Preventing the binding of RAS to the plasma membrane of cells could potentially cause inactivation. Farnesyltransferase, which may bind to RAS in cells, is growing in its popularity as an anticancer treatment [26,31–33]. Issues that are hindering the research progress in the field of RAS include the use of cell lines and mouse models to test RAS-inhibiting drugs. Mouse models do not provide an accurate representation of human cancer. Instead, organoids, which are small-scale tissues, can be used to study human tumors, as they are grown from cancer patients. Colorectal cancer organoid tissue can be used to test the effectiveness of drug combinations that inhibit effectors in the downstream RAS pathway. Research using organoids of *K-RAS*-related colon cancer showed that drug combination therapy of Afatinib and Selumetinib was partially successful in *K-RAS*-mutated colon cancer [34]. The tumor failed to grow due to cell cycle inhibition; however, the tumor cells did not die. This same combination tested on normal, nonmutated *K-RAS* organoid tissue yielded more successful results, with cell cycle arrest as well as tumor cell death [34]. RAS targeting is a continuing effort among researchers. The NIH has been focusing on RAS for the past few years [9]. The discoveries of genetic, genomic and clinical biomarkers have helped the development of personalized medicine [35]. Personalized medicine allows the treatment of disease by optimizing response to therapy and maximizes the therapeutic outcome with low or no toxicity. The identification of *K-RAS* as a clinical biomarker and potential therapeutic target has attracted the scientific community to develop an effective and precise anticancer drug. Inhibitors which block farnesylation of Ras have been developed or are under clinical trial studies [35]. Tipifarnib, approved by the US FDA for the treatment of elderly acute leukemia, is a Ras pathway inhibitor [35].

## Conclusion

The frequency of *RAS* mutations is among the highest for any gene in human cancers and development of inhibitors of Ras-mitogen activated protein kinase pathway as potential anticancer agents is a very promising pharmacologic strategy. Personalized medicine allows for the treatment of patient by optimizing therapeutic outcome and lowering toxicity. Personalized medicine is possible due to genetic, genomic and clinical biomarker development. The latest advances in the understanding of RAS biology have led to new opportunities for directly targeting RAS or to more effectively target key RAS effectors. While these new agents and approaches have already shown promising results in preclinical and clinical studies, the complexity of RAS signaling and the potential for robust adaptive feedback continues to present substantial challenges. Therefore, the development of targeted therapies will require a detailed understanding of the properties and dependencies of specific cancer to RAS mutation.

## Future perspective

Developments of newer cancer therapies require identification of novel targets. As personalized medicine allows treatment of the patient by optimizing the therapeutic outcome and lowering toxicity, further research in the area of *RAS* mutations will progress in the coming years.

**Executive summary**

- Prevalence of RAS (rat sarcoma virus) protein mutations has provided a popular target for the development of novel cancer therapeutics.
- Mutations in the RAS protein in the different types of cancers are discussed.
- Latest advances in the understanding of RAS biology have led to new opportunities for directly targeting RAS in the development of newer cancer therapeutics.
